# Comparative Analysis of Engineering Carbonation Model Extensions to Account for Pre-Existing Cracks

**DOI:** 10.3390/ma16186177

**Published:** 2023-09-12

**Authors:** Annika Lidwina Schultheiß, Ravi Ajitbhai Patel, Michael Vogel, Frank Dehn

**Affiliations:** Institute of Concrete Structures and Building Materials (IMB), Karlsruhe Institute of Technology (KIT), DE-76131 Karlsruhe, Germany; annika.schultheiss@kit.edu (A.L.S.); michael.vogel@kit.edu (M.V.); frank.dehn@kit.edu (F.D.)

**Keywords:** carbonation, crack, concrete, comparison, models

## Abstract

Cracks in reinforced concrete structures can accelerate the local depassivation of reinforcement due to carbonation. Different approaches have been proposed to account for pre-existing cracks within engineering models to predict the carbonation depth. In this study, we provide a detailed comparative analysis of different extensions available for the *fib* carbonation model to account for cracks, viz., crack influence factor (CIF) approaches, a diffusion-based model and the crack depth adaption. The model extensions are first validated against a dataset of lab data collected from the literature and additional experiments performed as the part of this study. The CIF approaches achieved the highest accuracy for the carbonation depth prediction when compared against lab data. The diffusion-based model was inaccurate for low ${\mathrm{CO}}_{2}$ concentrations. The crack depth adaption provides overly conservative results. No model was found to be best performing, and large scatter was observed between predicted and experimental values. This emphasizes the need for more detailed multi-physics-based models to achieve accurate predictions. For further comparison, service life predictions were conducted for two structural scales, viz., the whole structure and the cracked area. It is concluded that the choice of model extension and the structural scale of analysis have a large influence on predicted probability of failure.

## 1. Introduction

**Figure 1:**
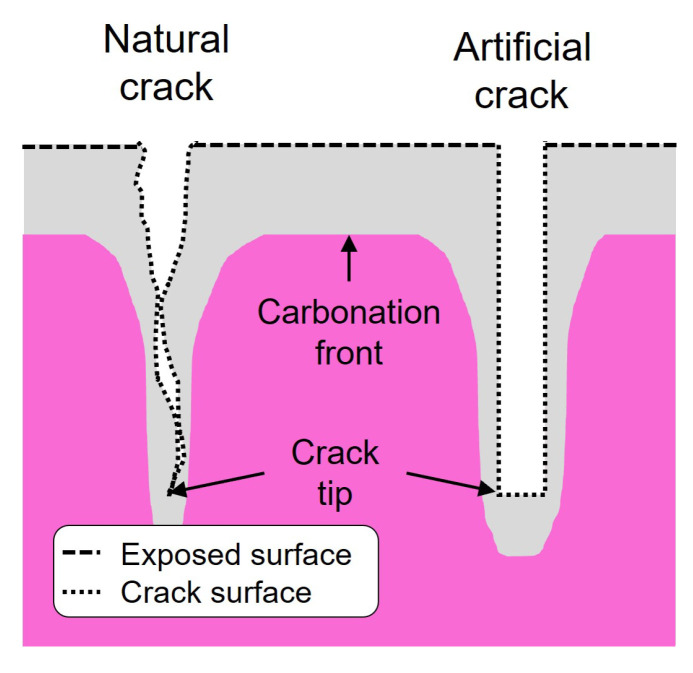
Carbonated concrete with two crack types: natural crack and artificial crack (notch).

The carbonation-induced depassivation of steel reinforcement is one of the key durability issues to be accounted for in models used for service life predictions of reinforced concrete structures. To predict the probability of depassivation, a model given in the *fib* Model Code for Service Life Design (*fib* MC SLD) [1] and the *fib* Model Code for Concrete Structures 2010 [2] (referred further on as the *fib* carbonation model) is often used in practice. This model is only applicable for uncracked concrete. However, the occurrence of cracks in loaded reinforced concrete structures is common [3,4] and sometimes even desirable to activate the stress transfer to the reinforcement [5]. Additionally, cracks can occur even in the absence of external load in concrete structures due to stresses induced by the heat of hydration and autogenous and drying shrinkage [4,6,7,8]. However, from a durability perspective, cracks create additional transport pathways that can accelerate the transport of ${\mathrm{CO}}_{2}$ in concrete, and an additional carbonation front can develop from the crack’s surface (see Figure 1). As a result of the presence of cracks, the carbonation front can reach the reinforcement faster than in uncracked concrete.

Experimental studies to investigate the influence of cracks are either conducted on artificial cracks [9,10,11,12] or on natural cracks [9,12,13]. Both crack types are shown in Figure 1. Artificial cracks are usually generated as notches, which have the same width along the crack path and a defined crack depth. In contrast, natural cracks have a realistic tortuosity and roughness along the crack path.

**Figure 2:**
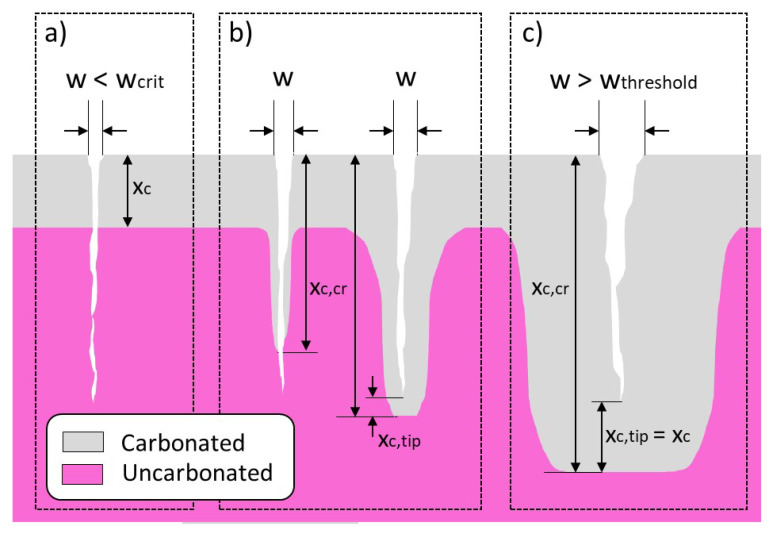
Schematic illustration of carbonation front in concrete with cracks depending on the surface crack width *w*, where $x_{c}$, $x_{c,tip}$ and $x_{c,cr}$ are the carbonation depth from the surface, from the crack tip and the carbonation depth at the location of a crack, respectively. (**a**) Crack width *w* smaller than critical crack width; (**b**) Crack width *w* between critical crack width and threshold crack width; (**c**) Crack width *w* exceeds threshold crack width.

Many experimental studies [9,12,13,14,15,16,17] have reported that carbonation rates in cracks increase with increasing crack width. Two limiting crack widths exist (see Figure 2). First is a critical crack width between 0.009 mm and 0.010 mm, below which cracks do not influence the carbonation [9,11] (Figure 2a), and second, a threshold crack width between 0.06 mm and 0.10 mm, above which the carbonation front from the crack surface and crack tip develops with the same rate as the carbonation front from the exposed concrete surface [9,10,11,12,13] (Figure 2c). Al-Ahmad et al. [9] reported that the critical crack width only applies for natural cracks and not for artificial cracks with smooth surfaces. Schiessel [18] theorizes that the carbonation depth in the cracked area is directly proportional to the square root of the crack width. However, Sullivan-Green et al. [19] state that they did not see such a $\sqrt{w}$-relationship in their experiments. Some studies argue that the crack depth is a more influential parameter than crack width [10,11,14,18].

Several engineering models that predict the carbonation depth at the location of a crack have been presented in the literature. These models need the carbonation depth of the uncracked concrete as an input parameter. Some engineering models are analytically based on the ${\mathrm{CO}}_{2}$ diffusion process in cracks [18]. Others use empirically derived functions based on the crack depth [12] or empirically derived crack influence factors (CIF) [14,20]. These *CIF*s are multiplied by the carbonation depth of the uncracked concrete to predict the carbonation depth at the location of a crack. All these engineering models are designed to deterministically predict the carbonation depth at the location of a crack. Nevertheless, a comparative analysis of these extensions and their impact in the context of probabilistic service-life assessment has not been critically evaluated. Therefore, it is unclear whether these engineering approaches result in comparable probabilities of reinforcement depassivation and if the consideration of cracks significantly impacts the result of service life predictions.

In this study, the *fib* carbonation model for uncracked concrete is extended using all existing approaches found in the literature to predict the carbonation depth at the location of a crack. Time-dependent changes in crack properties due to further damage or crack healing are ignored. The model extensions are compared against a wide range of experimental data collected from the literature and new experiments performed as a part of this study. Finally, the model extensions are used for the reliability assessment of an exemplary structure. Comparisons between models are made for both deterministic and probabilistic predictions. Probabilistic predictions are made for the cracked area and the whole structure because the probability of depassivation is vastly dependent on the structural scale considered for analysis in a reliability assessment. Finally, the limitations of these model extensions are critically discussed to explore further research directions and enable better predictions of the carbonation depth at the location of a crack.

## 2. Carbonation Models for Concrete

In this section, the *fib* carbonation model is recalled, and four extensions to account for cracks are presented. Finally, the methodology for applying these extensions in the context of service life predictions across different structural scales is presented.

### 2.1. Model for Uncracked Concrete

As mentioned previously, model extensions for cracked concrete require the carbonation depth of the uncracked concrete as an input parameter. In recent years, several models [21,22,23,24,25,26,27,28,29] to predict the carbonation depth of uncracked concrete have been introduced. In this study, the established *fib* carbonation model [1,2] is used (see Equation (1)). The *fib* carbonation model assumes a one-dimensional diffusion process (Fick’s first law) and is only valid for uncracked concrete with a constant diffusion coefficient. To predict the carbonation depth in a probabilistic manner, some input parameters are given in a normal distribution denoted as N(mean,std). For deterministic predictions, only the mean values are used. The carbonation depth of uncracked concrete $x_{c,uncr,fib}\left(t\right)$ [mm] can be predicted with

(1) $$x_{c,uncr,fib}\left(t\right)=\sqrt{2\times k_{e}\times k_{c}\times \left(k_{t}\times R_{Acc,0}^{-1}+\epsilon_{t}\right)\times C_{s}}\times \sqrt{t}\times W\left(t\right)$$

where $k_{t}=N\left(1.25,0.35\right)$ is a regression parameter; $R_{Acc,0}^{-1}$ [$\frac{{\mathrm{mm}}^{2}/\mathrm{year}}{{\mathrm{kgCO}}_{2}/m^{3}}$] is the inverse effective carbonation resistance of dry concrete, which is determined at a certain point in time ($t_{0}$); $\epsilon_{t}=N\left(315.5,48\right)$ is the error term; $C_{s}$ [$\frac{{\mathrm{kgCO}}_{2}}{m^{3}}$] is the ${\mathrm{CO}}_{2}$ concentration; and *t* [years] is the time. For the prediction of the carbonation depths from the laboratory experiments, $k_{t}=1$ and $\epsilon_{t}=0$ are used. The environmental function $k_{e}$ [−] is given as:

(2) $$k_{e}={\left(\frac{1-{\left(\frac{RH_{real}}{100}\right)}^{f_{e}}}{1-{\left(\frac{RH_{ref}}{100}\right)}^{f_{e}}}\right)}^{g_{e}}$$

with the relative humidity of the carbonated layer $RH_{real}$ [%], the reference relative humidity $RH_{ref}=65$% and the exponents $f_{e}=5$ and $g_{e}=2.5$. The execution transfer parameter $k_{c}$ [−], which considers the influence of the curing period on carbonation resistance, is given as:

(3) $$k_{c}={\left(\frac{t_{c}}{7}\right)}^{b_{c}}$$

with the period of curing $t_{c}$ [days] and the regression parameter $b_{c}=N\left(-0.567,0.024\right)$. The weather function W(t), which takes the meso-climatic boundary conditions due to raining events into account, is given as:

(4) $$W\left(t\right)={\left(\frac{t_{0}}{t}\right)}^{\frac{{\left(p_{SR}\times ToW\right)}^{b_{w}}}{2}}$$

where the probability of driving rain is $p_{SR}$ [−], ToW [−] $=\frac{days\;with\;rainfall\;\geq \;2.5\;mm\;per\;year}{365}$, the reference time of 28 days is $t_{0}=0.0767$ years, and the regression parameters are $b_{c}=N\left(-0.567,0.024\right)$ and $b_{w}=N\left(0.446,0.163\right)$.

### 2.2. Models for Cracked Concrete

The model extensions account for the influence of cracks based on the crack’s geometrical parameters, such as the crack’s width at the surface and the crack’s depth. The crack’s width at the surface is easy to determine through non-destructive measurements. Other crack properties, such as the crack’s depth, need destructive structural sampling for investigation. The influence of the damaged zone around a crack, which has an increased diffusion coefficient [30,31], is neglected in all engineering models.

#### 2.2.1. CIF Approach

Crack influence factors (CIF) are multiplied by the carbonation depth of the uncracked concrete to predict the carbonation depth at the location of a crack.

(5) $$x_{c,cr}=CIF\times x_{c,uncr,fib}\left(t\right)$$

where $x_{c,cr}$ [mm] is carbonation depth considering a crack. Kwon et al. [20] presented a CIF based on a field investigation of 27 reinforced concrete columns subjected to carbonation for eighteen years. Based on this investigation, the CIF(w) is a function of the crack width at the surface *w* [mm].

(6) $$CIF\left(w\right)=\left(2.816\sqrt{w}+1\right)$$

De Schutter [14] presented a CIF as a function of the crack width *w* [mm] and the crack depth [mm] based on accelerated laboratory experiments with artificial cracks. It was verified statistically that the CIF(w,d) is independent of the mortar composition and treatment. Therefore, the CIF(w,d) can be used for the carbonation predictions of any cracked concrete. To account for the statistical variation in the CIF(w,d), a coefficient (λ) with a log-normal distribution logN(1,0.3) is introduced.

(7) $$CIF\left(w,d\right)=\lambda \times e^{\left(0.4376\times d^{0.3426}\times w^{0.476}\right)}$$

For further discussion, we refer to approaches with the CIF of Kwon et al. [20] and the CIF of de Schutter [14] in combination with Equation (5) as CIF(w) and CIF(w,d), respectively.

#### 2.2.2. Diffusion-Based Model

Schiessl [18] modeled the carbonation process in a crack with Equation (8), considering the diffusion of ${\mathrm{CO}}_{2}$ from the air into the crack and the diffusion of ${\mathrm{CO}}_{2}$ into the concrete at the crack surfaces. The model given in Equation (8) predicts the carbonated crack depth in dry indoor climates [18,32] and assumes that the carbonation front in the crack is located where the ${\mathrm{CO}}_{2}$ concentration equals zero. The model should not be used for uncracked concrete (w=0 mm) because this results in an unrealistic carbonation depth of $x_{c}=0$ mm.

(8) $$x_{c,cr,Schiessl}\left(t\right)=\sqrt{D_{crack}\times w\times \sqrt{\frac{4\times C_{s}\times t}{D_{cc}\times a}}}$$

where $D_{crack}=4.41504$×$10^{8}$$\frac{{\mathrm{mm}}^{2}}{\mathrm{year}}$ and $D_{cc}$ [$\frac{{\mathrm{mm}}^{2}}{\mathrm{year}}$] are the diffusion coefficient of ${\mathrm{CO}}_{2}$ in the crack and of carbonated concrete, *w* [mm] is the crack width, $C_{s}$ [$\frac{{\mathrm{kgCO}}_{2}}{m^{3}}$] is the ${\mathrm{CO}}_{2}$ concentration, and *a* [$\frac{{\mathrm{kgCO}}_{2}}{m^{3}}$] is the ${\mathrm{CO}}_{2}$ binding capacity of the concrete, which is given in [33] as

(9) a=0.589×C×CaO×α

where *C* [$\frac{\mathrm{kg}}{m^{2}}$] is the cement content, CaO [−] is the calciumoxide content in cement, and α [−] is the degree of hydration. The diffusion coefficient $D_{cc}$ [$\frac{{\mathrm{mm}}^{2}}{\mathrm{year}}$] is computed with the following relationship given in [33]:

(10) $$D_{cc}=R_{Acc,0}^{-1}\times a$$

In this study, the model of Schiessl is further extended for cracks with a finite crack depth *d*. Equation (11) is used to calculate the carbonation progress in the crack until the carbonation front reaches the crack tip; any effects due to rain and relative humidity changes are neglected. After the carbonation front reaches the crack tip ($t=t_{tip}$), the carbonation continues through the concrete. It is assumed that the diffusion properties of the concrete underneath the crack tip are equal to the diffusion properties of the uncracked concrete. This results in the same equation as that in the *fib* carbonation model. Therefore, in this study, once the carbonation depth reaches the crack tip, the *fib* carbonation model, considering the relative humidity changes, is used to compute the carbonation front progress. This approach is summarized as follows:

(11) $$x_{c,cr,dif-based}\left(t\right)=\left\{\begin{array}{cc} \sqrt{D_{crack}\times w\times \sqrt{\frac{4\times C_{s}\times t}{D_{cc}\times a}}}, & \mathrm{if}\;t\leq t_{tip}\\ d+x_{c,uncr,fib}\left(t-t_{tip}\right), & \mathrm{if}\;t>t_{tip} \end{array}\right.$$

where the time when the carbonation front reaches the crack tip $t_{tip}$ [year] is calculated as:

(12) $$t_{tip}=\frac{d^{4}\times D_{cc}\times a}{4\times D_{crack}^{2}\times w^{2}\times C_{s}}$$

The approach presented in Equation (11) is referred to in further discussion as the diffusion-based model.

#### 2.2.3. Crack Depth Adaption

Bogas et al. [12] present an empirical model (see Equation (13)) to predict the carbonation depth at the location of a crack under real exposure conditions as a function of the crack depth *d*. The model assumes that the carbonation depth always exceeds the crack depth [12]. One drawback of this model is that it requires the carbonation coefficients ($K_{cr}$ and $K_{uncr}$) for both cracked and uncracked areas, which are obtained through laboratory experiments with increased ${\mathrm{CO}}_{2}$ concentrations ($C_{s,lab}$).

(13) $$x_{c,cr,Bogas}\left(t\right)=d+K_{uncr}\times \sqrt{\frac{C_{s}}{C_{s,lab}}}\times \sqrt{t}-\frac{K_{uncr}\times d}{K_{cr}}$$

In this study, their approach is simplified with the conservative assumption that the crack does not offer any resistance against carbonation. This corresponds to the carbonation process in cracks where the critical crack width is exceeded. Therefore it is assumed that the carbonation front at the crack tip progresses at the same rate as the carbonation front developing from the surface into the uncracked concrete. For the prediction of the carbonation depth in uncracked concrete, the *fib carbonation model* is used. This approach is described in Equation (14) and is referred to as the *crack depth adaption*.

(14) $$x_{c,cr,d_{cr}}\left(t\right)=d+x_{c,uncr,fib}$$

### 2.3. Probabilistic Analyses for Concrete with Pre-Existing Cracks

The probability of depassivation $p_{dep}$ at the aimed service life $t_{SL}$ [years] can be calculated with probabilistic approaches such as the Monte Carlo simulation. The limit state function (Equation (15)) to be evaluated is in accordance with the *fib* MC SLD [1].

(15) $$p_{dep}=p\left\{c_{nom}-x_{c}\left(t_{SL}\right)\right\}<p_{0}\left(t_{SL}\right)$$

where $c_{nom}$ [mm] is the nominal concrete cover and $x_{c}\left(t_{SL}\right)$ [mm] is carbonation depth, which can be computed with all modeling approaches presented in Section 2.1 and Section 2.2. The required reliability criteria $p_{0}\left(t_{SL}\right)\approx 10\%$ equals the recommended minimum reliability index of β=1.3 for the limit state of carbonation [1].

**Figure 3:**
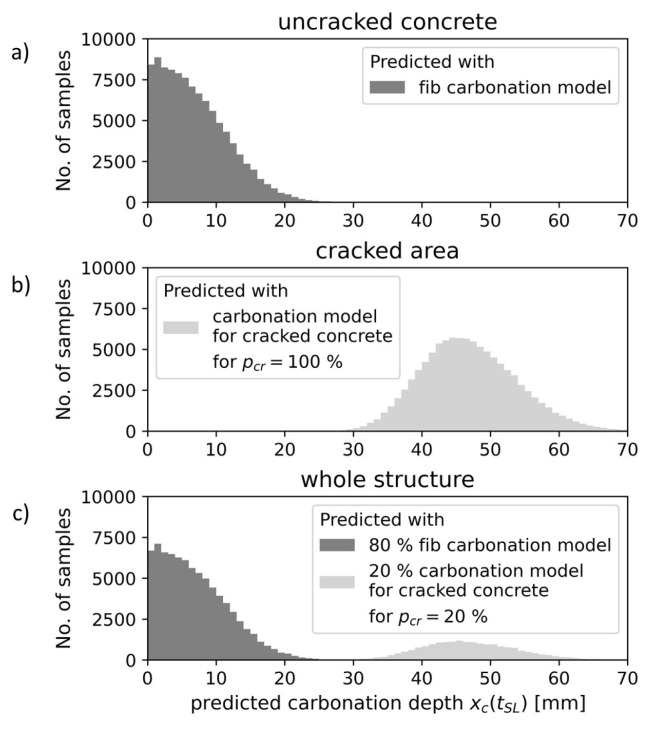
Structural scales of reliability analyses: (**a**) Uncracked concrete; (**b**) Cracked area; (**c**) Whole structure. For the Monte Carlo simulation to predict 100,000 carbonation depths $x_{c}\left(t_{SL}\right)$, different models are used. Here, the crack depth adaption was used as a carbonation model for cracked concrete.

According to Schultheiß et al. [34], reliability analyses can be performed for uncracked concrete, for the cracked area of concrete, or for the entire concrete structure, which includes a small portion of the cracked surface. Figure 3 illustrates the predicted carbonation depths for each structural scale. Conducting a reliability analysis for the cracked area (Figure 3b), the probability of carbonation-induced depassivation is predicted using a model for cracked concrete. The increased carbonation depths in the cracked area result in a higher probability of depassivation compared to uncracked concrete. The reliability analysis for the whole structure (Figure 3c) gives a general probability of the carbonation-induced depassivation of the reinforcement if the concrete cover includes some cracks. In the latter approach, only an equivalent to the fraction of cracked surface $p_{cr}$ of the carbonation depths is predicted with a model for cracked concrete; the rest is predicted with the *fib* carbonation model for uncracked concrete.

## 3. Experimental Studies

In this section, a large dataset compiled from the literature encompassing experimental studies that measure carbonation depth for paste, mortar, and concrete with pre-existing cracks is first discussed. Based on the literature review, a data gap has been identified, and an additional experimental study on mortar specimens with artificial cracks has been conducted, which is further discussed in this section.

### 3.1. Validation Data from Literature

Many experimental studies report carbonation depths in cracked concrete [10,12,13,14,15,16,17,19,35,36,37]. Studies with missing information regarding crack properties or mix design [15,17,19,35,36] and studies with specialized mix designs such as [12,16] were excluded from the dataset. The carbonation depths at the location of the cracks were collected from [10,13,14,37], which includes studies on both natural cracks and artificial cracks created as notches. A summary of this literature data is given in Table 1. The dataset includes carbonation depths at the location of cracks from paste, mortar, and concrete samples with various replacement rates of ordinary Portland cement (OPC) to fly ash. The accelerated testing conditions vary between 1 and 20 vol.% ${\mathrm{CO}}_{2}$ concentration.

The inverse carbonation resistance parameter $R_{ACC}^{-1}$ for the uncracked concrete is calculated with Equation (16). According to the *fib* MC SLD [1], the $R_{ACC}^{-1}$ should be based on exposure of 2 vol.% ${\mathrm{CO}}_{2}$ at 28 days. Therefore, for the collected data, $R_{ACC}^{-1}$ is calculated with the ${\mathrm{CO}}_{2}$ concentration given in Table 1 and the carbonation depth $x_{c,uncr}\left(t\right)$ determined for *t* closest to 28 days of ${\mathrm{CO}}_{2}$ exposure.

(16) $$R_{ACC}^{-1}=\frac{x_{c,uncr}^{2}}{2\times k_{e}\times k_{c}\times C_{s}\times t}$$

The parameters $k_{c}$ and $k_{e}$ given in Table 1 are based on the curing time and the relative humidity of the experimental conditions of each study. If the CaO content was not given, it is assumed that OPC is 57.9 w.% and fly ash is 3.14 w.%, which corresponds to the materials given in Table 2.

The maximum degree of hydration α, needed for the diffusion-based model was computed according to [38] with

(17) $$\alpha =0.239+0.745\times tanh\left(3.62\times \left(\frac{w}{b}\right)-0.095\right)$$

The deterministic prediction of the carbonation depth is conducted using the *fib* carbonation model (Equation (1)) and four model extensions. The CIF approaches include CIF(w) (Equations (5) and (6)) and CIF(w,d) (Equations (5) and (7)), the diffusion-based model (Equation (11)) and the crack depth adaption (Equation (14)). All input parameters needed to predict the carbonation depth at the location of the crack are summarized in Table 3.

### 3.2. Experimental Study

The dataset collected from the literature does not include data from experimental conditions, which corresponds to the required conditions to determine the $R_{ACC}^{-1}$ according to the *fib* MC SLD. To address this limitation, an additional experimental study was conducted. Furthermore, the dataset from the literature has limited variation in crack depth under low ${\mathrm{CO}}_{2}$ exposure. Therefore, in this study, artificial cracks were created as notches to ensure good control of the crack depth. Two depths, 10 and 20 mm, were investigated.

Mortar prisms with the dimensions 40 mm × 40 mm × 160 mm were produced in accordance with DIN EN 196-1 [39]. For the mortar, the binder-sand-water ratio was 1:2:0.5. Portland cement and fly ash were used as binders. Samples made with Portland cement (CEM I 42.5 R) were labeled OPC, while samples with the blended binders (80 w.% CEM I, 42.5 R, 20 w.% fly ash) were labeled OPC + FA. The clinker composition of the binders is given in Table 2.

The cracks were created as notches similar to that used in other studies [10,14,37]. Thin steel sheets were placed in the fresh mortar and removed after 4 h to create notches. The use of mortar simplifies the precise placing of the steel sheets and thus improves the chances of creating a crack with a constant crack width along the crack depth. For each mortar, 12 specimens were cast, viz., 4 without cracks and 8 with artificial cracks, having a unique combination of crack width, crack depth, and crack distance. The aimed crack widths of about 0.10 and 0.15 mm were confirmed using a digital optical microscope. For each crack width, two samples with a 10 mm crack depth and two samples with a 20 mm crack depth were produced. For each crack depth and crack width combination, the distance between the cracks for one sample was 20 mm with 5 cracks, and for the other, it was 40 mm with 3 cracks. The crack depth of 20 mm is the minimum value of the concrete cover according to Eurocode 2 [5]. The 40 mm distance between the cracks corresponds to a plausible crack spacing that can be obtained under certain conditions when determining the theoretical maximum crack width in the serviceability limit state, according to Eurocode 2 [5].

**Figure 4:**
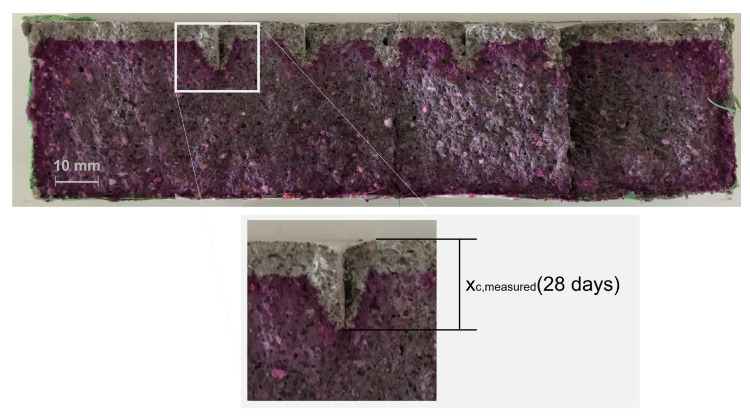
Specimens’ half with carbonation front, with focus on the carbonation depth at a crack. OPC + FA sample: *w* = 0.1 mm, *d* = 10 mm, crack distance = 20 mm.

Apart from the exposed surface, other sides of the sample were coated with a flexible high-performance sealing tape before the samples were placed into the carbonation chamber. In addition to this, the carbonation test was conducted according to the *fib* MC SLD [1] with exposure to 2 vol.% ${\mathrm{CO}}_{2}$ for 28 days at 20 ± 2 °C and 65 ± 5% RH. To measure carbonation depth, the specimens were split into two halves. Both sides were sprayed with an indicator solution consisting of 1 g phenolphthalein per liter. The measurement of the carbonation depths was performed with a caliper gauge that has an accuracy of 0.01 mm. One half of the carbonated sample for a sample specimen is shown in Figure 4. For each mix, 56 measurements of the carbonation depth of uncracked concrete and 16 measurements of the carbonation depth for each combination of crack width and crack depth were taken.

**Figure 5:**
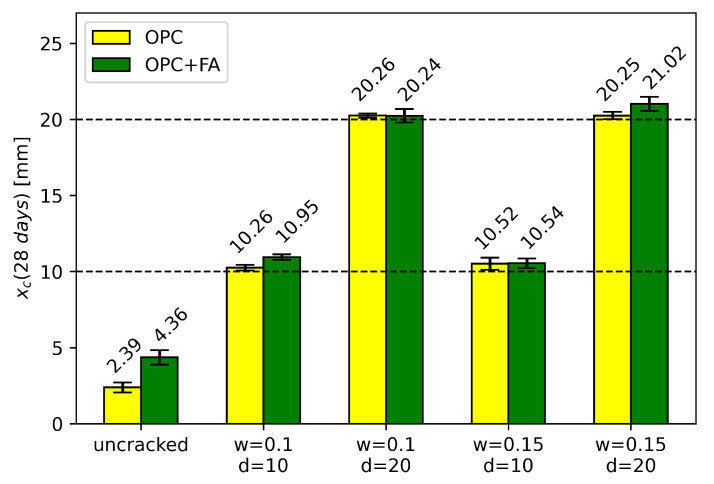
Mean values and standard deviation of measured carbonation depths after 28 days for uncracked concrete (each 56 measurements) and at the location of cracks (each 16 measurements) with different crack widths *w* [mm] and crack depths *d* [mm] for mixes OPC and OPC + FA. The crack depths *d* are indicated with horizontal lines.

Figure 4 shows that the carbonation depth measured from the crack tip was smaller than the carbonation depth perpendicular to the crack surface. Additionally, the carbonation depth perpendicular to the crack surface was smaller than the carbonation depth from the exposed surface. These results are consistent with the results from [37]. The average carbonation depths of the uncracked specimens and the locations of the cracks are given in Figure 5. Investigating the carbonated area around a crack, no interaction with the carbonation front of another crack was found. Either the test duration was too short or the crack distance was too large. In Figure 5, the cracks are therefore solely characterized by the crack width and crack depth.

All cracks were fully carbonated, which is consistent with previous notch studies [10,12]. Moreover, the carbonation front was, on average, located between 0.25 and 1.02 mm below the crack tip. Figure 5 shows that an increase in the crack width did not necessarily lead to greater carbonation depths. This is in accordance with [10], where little variation in carbonation depth across the 0.10 to 0.61 mm crack width range was reported. On the other hand, there are studies on artificial cracks that show a larger influence of the crack width on the carbonation depth [14,15,17,36]. Mullem et al. [13] further found that the influence of crack width is more pronounced for natural cracks compared to artificial cracks due to the crack tortuosity effect. In the case of the present study, the cracks had a rather low tortuosity, which is a consequence of the technique used to create notches.

All input parameters needed to predict the carbonation depth for the models at the location of the crack are summarized in Table 4. For the OPC and OPC + FA samples, $R_{ACC,0}^{-1}$ was calculated using the carbonation depth of the uncracked concrete.

## 4. Deterministic Evaluation of Models against Experimental Datasets

In this section, the models are evaluated against the dataset described in Section 3. First, the *fib* carbonation model (Section 2.1) is validated with carbonation depths measured from uncracked specimens. Then, the four model extensions that account for cracks (Section 2.2) are evaluated using the carbonation depths at the location of the cracks. Influencing factors such as the crack properties, the crack type, the mix design, and the exposure conditions are discussed.

### 4.1. Uncracked Concrete

**Figure 6:**
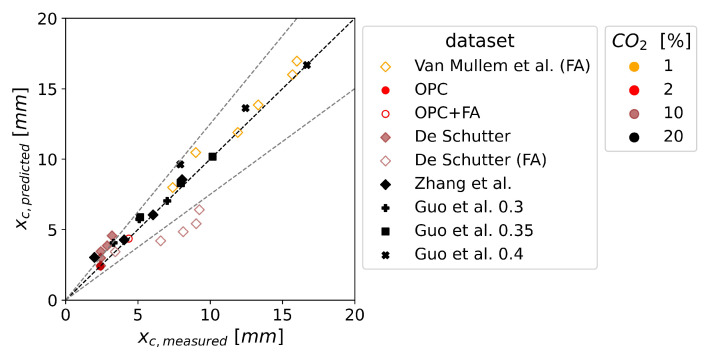
Deterministic prediction of the carbonation depth $x_{c}$ for the uncracked concrete with the *fib carbonation model* depending on the ${\mathrm{CO}}_{2}$ concentration (see color). Fly ash mixes are indicated with a white face color. $x_{c,measured}$ from own experiments and [10,13,14,37]. Note: Perfect predictions were gained for all $x_{c,measured}$ used to calculate $R_{ACC}^{-1}$.

Figure 6 illustrates the predicted carbonation depth of uncracked concrete using the *fib* carbonation model, as well as the derivation of the 25% accuracy level. In general, the predictions of the carbonation depths were mostly accurate. The underestimation of De Schutter’s fly ash mix tends to increase with larger carbonation depths, indicating a reduced accuracy for longer exposure times. In general, the prediction accuracy seemed to be independent of the ${\mathrm{CO}}_{2}$ concentration in the laboratory experiments. It has to be noted that Zhang et al. investigated concrete and Guo et al. paste, whereas all other studies investigated mortar. However, no influence on the prediction accuracy due to these differences in mix design can be found in Figure 6.

### 4.2. Cracked Concrete

**Figure 7:**
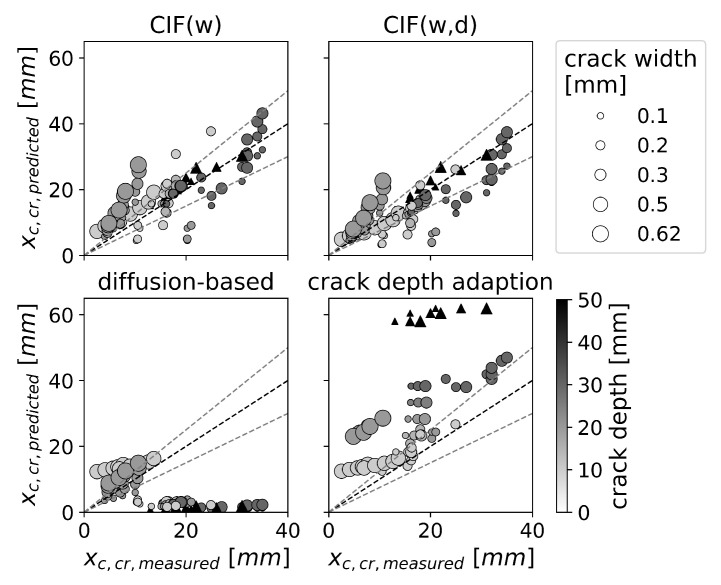
Effect of crack width (marker size) and crack depth (color) on the accuracy of predictions for carbonation depth at the location of a crack $x_{c,cr}$, for natural cracks (represented by ∆) and artificial cracks (represented by ∘). The gray lines indicate a 25% margin of prediction error.

Figure 7 shows the comparison between predicted and experimental carbonation depth for cracked concrete. The model with the best prediction accuracy was CIF(w,d). However, it should be noted that this model was derived using parts of the validation data. In contrast, CIF(w), which was derived from structural sampling, had a similar prediction accuracy to that of CIF(w,d). For both CIF models, the prediction uncertainty was ±20 mm and seems to be independent of the crack width or crack depth. The crack depth adaptation overestimates the majority of carbonation depths, while the prediction accuracy tends to decrease with increasing crack depth. The diffusion-based model tends to underestimate the carbonation depth with crack widths smaller than 0.3 mm. Furthermore, it underestimates all cracks with crack depths greater than 25 mm.

Figure 7 highlights the significant influence of the crack type on the prediction accuracy. As discussed in Section 3.2, the prediction accuracy for artificial cracks seemed to be strongly dependent on the crack depth. This is in line with the findings of Figure 7, which shows that the crack depth adaptation that accounts for crack depth provides a better approximation for artificial cracks than for natural ones.

**Figure 8:**
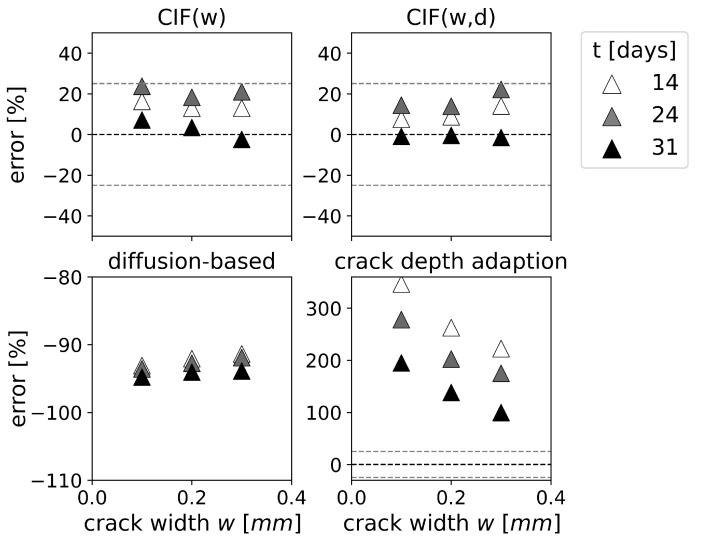
Time-dependent prediction error $\left(=\frac{x_{c,predicted}-x_{c,measured}}{x_{c,measured}}\times 100\;\%\right)$ for natural cracks as function of the crack width *w*. Experiments from van Mullem [13] (1% ${\mathrm{CO}}_{2}$, *d* = 50 mm). Note different *y*-axis scales.

For natural cracks, Figure 8 displays the time-dependent prediction error [%] for each model. The crack depth adaptation can overestimate the carbonation depth of natural cracks up to 346%. In fact, the predicted carbonation depths for the natural cracks even exceed the specimen height. This could lead to the conclusion that the crack depth adaptation may not be appropriate for real structures. However, Figure 8 also shows that the overestimation of natural cracks in the case of the crack depth adaptation decreases with increasing crack width, and the prediction accuracy significantly improves with increasing exposure time. The CIF approaches outperform the crack depth adaptation, which indicates that the carbonation depth for natural cracks is primarily dependent on the crack width. This is contradictory to the observations made for artificial cracks. The CIF approaches have a prediction error for natural cracks less than 25%. However, at 31 days exposure, the CIF approaches start underestimating the carbonation depth. The diffusion-based model significantly underestimates the carbonation depth of natural cracks. Furthermore, the underestimation increases with increasing exposure time, as illustrated in Figure 8.

**Figure 9:**
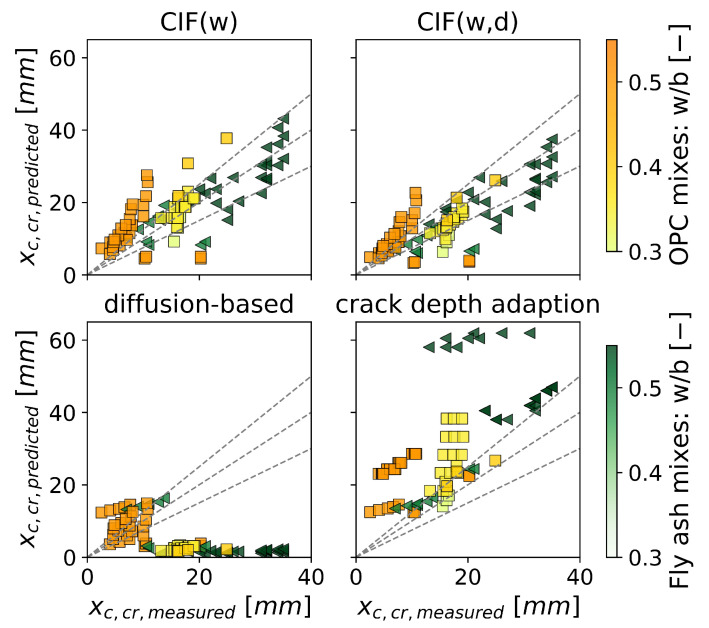
Effect of mix design and water-binder ratio on prediction accuracy of carbonation depth at the location of a crack $x_{c,cr}$. OPC mixes (◃, yellow), fly ash mixes (□, green).

Figure 9 shows the model predictions against experimental data for different mix designs. For the CIF approaches and the crack depth adaptation, the mix design is accounted for with the parameter $R_{ACC}^{-1}$ in the *fib* carbonation model. However, these models do not consider any other potential effects that the mix design may have on the crack. Figure 9 shows that CIF approaches tend to overestimate the carbonation depth of the OPC mixes with increasing w/b-ratio. The carbonation depth of fly ash mixes is prone to be underestimated with the CIF approaches. The crack depth adaptation overestimates carbonation depths for a w/b ratio greater than 0.3 for all mixes irrespective of binder replacement level with fly ash. The diffusion-based model considers the amount of material that can carbonate as an input parameter, but it does not improve the prediction accuracy. Both fly ash and OPC mixes are underestimated, especially for low w/b ratios.

**Figure 10:**
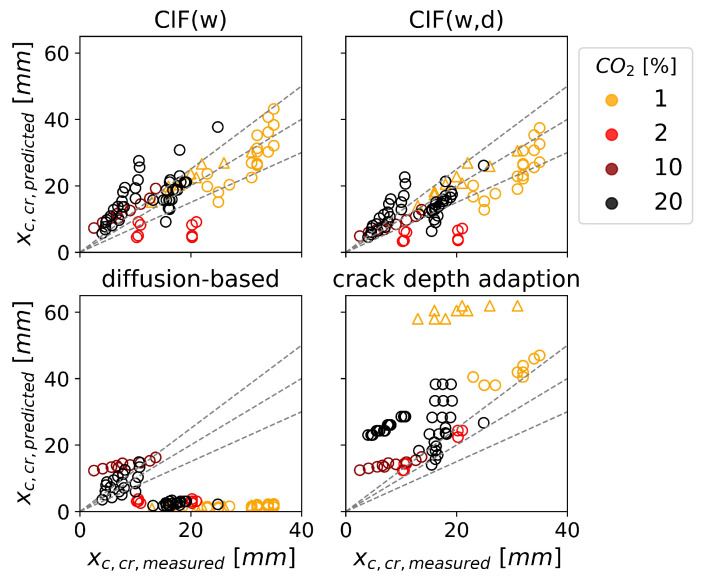
Influence of ${\mathrm{CO}}_{2}$ concentration on the prediction accuracy of the carbonation depth at the location of a crack ($x_{c,cr}$) for natural cracks ∆ and artificial cracks ∘. (Please refer to the colored version of the image).

Figure 10 shows the prediction accuracy for cracked concrete for different ${\mathrm{CO}}_{2}$ concentrations used in laboratory experiments. Prediction accuracy is found to be dependent on ${\mathrm{CO}}_{2}$ concentration even though all models account for it. This is contrary to that observed for uncracked concrete, as discussed in Section 4.1. CIF(w) tends to overestimate the carbonation depth for high ${\mathrm{CO}}_{2}$ concentrations; for CIF(w,d), this is less pronounced. The diffusion-based model underestimates the carbonation depth for 1 to 2% ${\mathrm{CO}}_{2}$ concentration. For the crack depth adaption, no trend in dependence on ${\mathrm{CO}}_{2}$ concentration was found.

## 5. Case Study for Service Life Predictions

In this section, the *fib* carbonation model (given in Section 2.1) and the four modeling approaches for cracked concrete (given in Section 2.2) are applied to a theoretical case study. The theoretical case study focuses on the cracked surface in the tension zone of the beam made with 100% OPC and a w/b-ratio of 0.5. The environmental conditions represent the climate of Karlsruhe/Rheinstetten, Germany. The climate data were provided by the Climate Data Center (CDC) Station 4177 [40]. The number of rainy days ToW was calculated as an average from the years 2010 to 2020. The distribution of the relative humidity RH is fitted on hourly measurements between December 2021 and December 2022. All input parameters are summarized in Table 5. To investigate the influence of the crack width and crack depth, deterministic calculations are presented in Section 5.1 using the mean values from Table 5. For the probabilistic calculations in Section 5.2, the crack width distribution *w* is chosen to fulfill the requirements for the maximum crack width of the Exposure Classes XC2, XC3, XC4 of Eurocode 2 [5]. The latter assumes that stress in the tension zone is transferred via the reinforcement because cracks can cut through the whole tension zone. Therefore, the mean value of the distribution of the crack depth *d* was chosen to equal the concrete cover $c_{nom}$.

The probability of carbonation-induced depassivation after 50 years is evaluated with the limit state equation (Equation (15)) by a Monte Carlo simulation with 100,000 samples. The reliability assessment is conducted for two different structural scales: First for the cracked area, and second for the whole structure, which includes $p_{cr}=0.65\%$ of the cracked surface. This fraction of the cracked surface equals an average crack spacing $s_{r,max}$ of about 3.8 cm for a crack width of about 0.25 mm designed according to Eurocode 2 [5].

### 5.1. Deterministic Results

**Figure 11:**
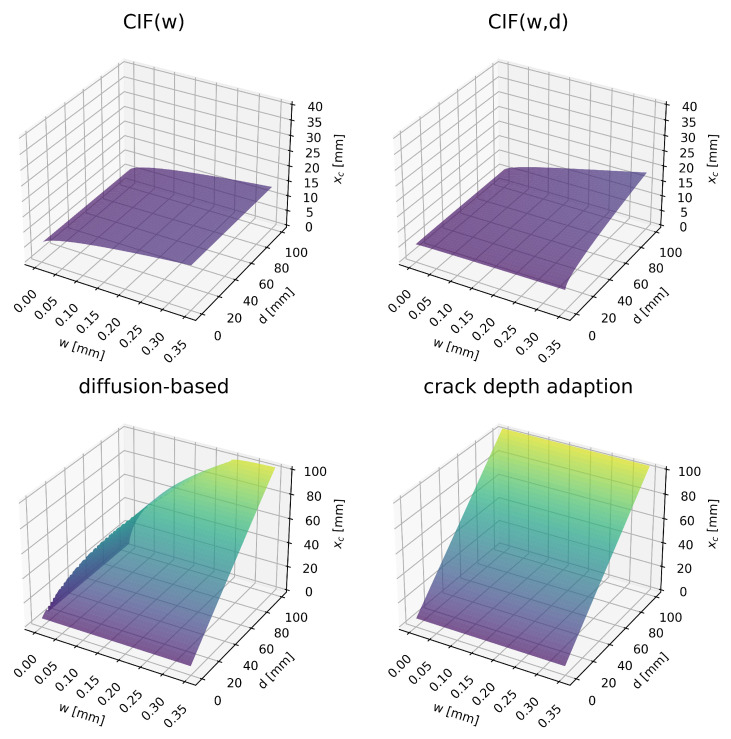
Carbonation depth $x_{c}$ after 50 years for different crack depths *d* and crack widths *w*. Predicted with mean values from Table 5.

Figure 11 illustrates the dependency of the carbonation depth on the crack width *w* and crack depth *d* after 50 years. The model extensions do not consider a limiting threshold or critical crack width below which carbonation depth is not influenced by crack width, even though those limits are commonly encountered in experimental investigations (as discussed in Section 1). For the investigated crack width (0.25 mm) and crack depth (35 mm) combination for the theoretical case study, the CIF approaches predict carbonation depths between 11 to 12 mm, while the diffusion-based model and the crack depth adaption predict carbonation depths of about 40 mm. For wide and deep cracks (*w* = 0.35 mm, *d* = 100 mm) the prediction results diverge even more. The CIF approaches predict carbonation depths in the range of 13 to 18 mm, which are significantly lower than the crack depth. In contrast, the other approaches predict carbonation depths in the range of 103 to 105 mm, which exceed the crack depth. For very fine cracks, the latter models also diverge significantly. For a fine and deep crack (*w* = 0.05 mm, *d* = 100 mm), the diffusion-based model predicts 44 mm and the crack depth adaption predicts a 105 mm carbonation depth. This shows that depending on the crack dimensions considered, the models can lead to inconclusive predictions.

### 5.2. Probabilistic Results

**Figure 12:**
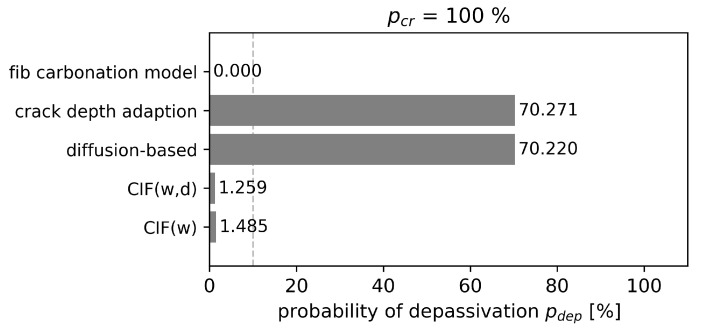
Probability of depassivation $p_{dep}\left(t_{SL}\right)$ of the cracked area in comparison to reliability criteria of 10%.

The 0% probability of depassivation after 50 years for the uncracked concrete is significantly lower than the required reliability criteria of $p_{0}\left(t_{SL}\right)\approx 10\%$. This is due to the fact that the concrete cover depth was selected taking into account the right exposure class according to Eurocode 2 [5]. Alongside the results of the uncracked concrete, the probability of depassivation of the cracked area is presented in Figure 12. As expected, the results of the reliability analysis are inconclusive. The CIF approaches CIF(w) and CIF(w,d) give similar results in the range of 1.3% to 1.5% depassivation probability and fulfill the reliability criteria of 10%. But, from the comparison with the lab experiment presented in Section 4.2, it is known that the CIF approaches tend to underestimate carbonation depths of natural cracks with increasing exposure time. Also, for this theoretical case study, as the mean crack depth is equal to the cover depth, it is very likely that at a service life of 50 years, the depessivation of steel reinforcement has occurred. Therefore, it seems plausible that the probability of depassivation is also underestimated by CIF approaches. The probability of depassivation for the other models is much higher. The crack depth adaption and the diffusion-based model predict a probability of depassivation of about 70%. Although the validation with the lab experiment showed that the diffusion-based model tends to underestimate the carbonation depth for low ${\mathrm{CO}}_{2}$ concentrations, the depassivation probability is similar to the crack depth adaption. In contrast, the crack depth adaption was the only conservative approach that largely overestimated the carbonation depths for the validation data. Nonetheless, after 50 years, the probability of depassivation is similar.

**Figure 13:**
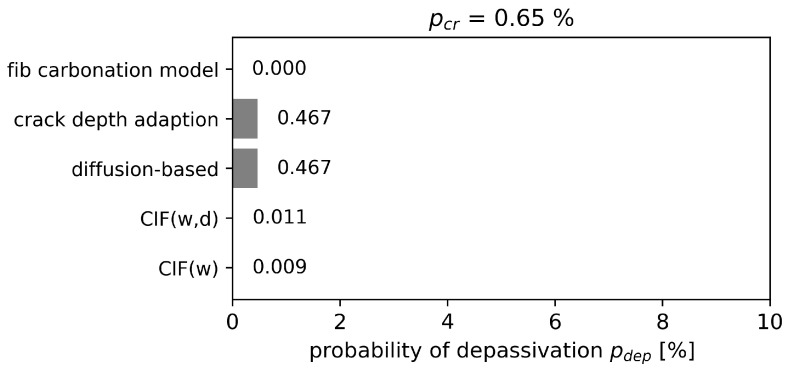
Probability of depassivation $p_{dep}\left(t_{SL}\right)$ of the whole structure with 0.65 % cracked area.

Evaluating the whole structure, it was assumed that 0.65% of the concrete surface is covered with cracks. The probability of depassivation for the whole structure is presented in Figure 13. Depending on the model, the results are one to three orders of magnitude apart. However, for all models, the probability of depassivation is significantly below 1% and therefore also significantly lower than the required reliability criteria of 10%. This is rather expected as cracks account for only a small fraction of the samples for service life predictions of whole structures. Closer evaluation of the probability of depassivation shows that nearly 70% of the crack location undergoes depassivation for crack depth adaptation and the diffusion-based model, which is consistent with the study of probability of depassivation focusing on cracked area.

## 6. Limitations of the Extensions for Carbonation-Induced Depassivation of Cracked Concrete

**Figure 14:**
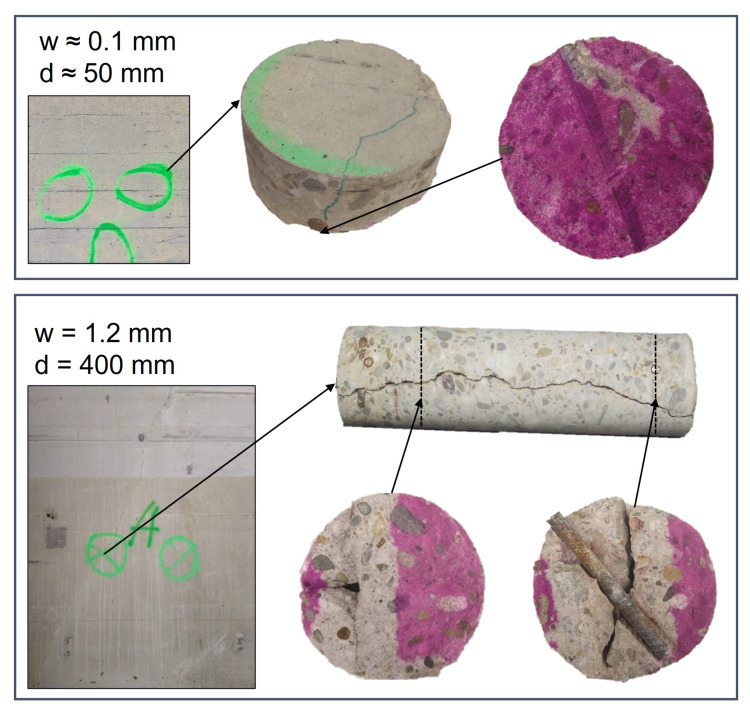
Carbonation front in the area of a crack after 56 year in an outer wall. $c_{nom}\geq$ 40 mm, wall thickness = 40 cm, dill-core diameter = 100 mm, constructed 1961–1965. (Source: first author).

The model validation demonstrated that the examined model extensions are only partly accurate in terms of predicting the carbonation of cracked concrete under the investigated laboratory conditions. The crack depth adaptation (nearly) strictly overestimated the carbonation depth at the location of a crack. On the other hand, other model extensions often underestimated the latter. The depassivation probability of about 1% in the cracked area as predicted by CIF approaches seems to be unrealistic low. This assertion gains further support from an exemplary investigation of the carbonation depth of an approximately 56 year old reinforced concrete wall shown in Figure 14. The exact time of the formation of the cracks is unknown. However, due to the extent of the cracks in the structure, it is likely that they occurred later during service life. The investigation showed that depassivation of the reinforcement occurred for cracks with surface crack widths between 0.1 mm and 1.2 mm. In the theoretical case study, the mean crack width was 0.25 mm and therefore significantly wider than the carbonated 0.1 mm wide crack.

For the whole structure, the probability of depassivation was for all model extensions below 1%. This could provide the misleading conclusion that the influence of cracks on the depassivation of the reinforcement is limited and can be ignored. However, localized failure of the reinforcement can lead to the collapse of the entire structure. For the investigation of the whole structure, only the fraction of the cracked surface was considered to be influenced by the crack. However, the area of carbonated concrete could be significantly higher. Investigations of cracked concrete (e.g., Figure 4 and Figure 14) confirm that the carbonation process in cracked concrete is a multi-dimensional process. The carbonation front does not just develop below the crack tip, but also from the crack surfaces. Therefore, the effect of cracks on the depassivation is not limited to the exact location of the crack but includes the carbonation front developing from the crack surface. As shown in Figure 14, the time-dependent development of this carbonation front perpendicular to the crack is again dependent on the crack properties such as the crack width and depth. However, the development of the carbonation front from the crack surface was neglected when investigating the whole structure including cracks.

Load-induced cracks that reach the reinforcement can damage the interface between the steel and the concrete. Several experiments [9,41,42,43] have shown that the carbonation front can extend from the crack to the damaged steel–concrete interface, resulting in a larger area with depasssivated reinforcement than that exposed by crack. Al-Ahmad et al. [9] found that micro-cracks in the steel–concrete interface of a width between 12 and 28 μm are sufficient to observe such effects. However, when investigating the whole structure including cracks, the development of the carbonation front along the reinforcement was neglected.

It is also questionable if the depassivation of the reinforcement due to carbonation is the suitable limit state for the reliability analysis of cracked concrete. Carevic et al. [36] argue that carbonation-induced depassivation cannot be considered a serviceability limit state because reinforcement corrosion rapidly occurs after just a few years in the case of cracked reinforced elements. It is generally recognized that cracks provide an additional path to the steel–concrete interface, creating favorable conditions for corrosion. In Figure 14, for the crack width of 1.2 mm, corrosion products can be found on the depassivated reinforcement. However, no corrosion-induced cracking and spalling and no significant loss of cross-section was observed. Al-Ahmad et al. [9] also observed the formation of small amounts of oxides on the steel surface in the area of the carbonated steel-concrete interface during carbonation tests. Corrosion experiments on carbonated specimens with cracks from Ghantous et al. [43], however, showed that the corrosion products did not spread beyond the limits of the carbonated (and therefore depassivated) length of the reinforcement. As a conclusion, they state that cracks of even 500 μm in width would not lead to a dangerous corrosion state. L’Hostis [44] also investigated the corrosion kinetics of carbonated reinforced cracked specimens that were exposed to wet–dry cycles. She reports that the carbonated length of reinforcement remained unchanged during her corrosion experiments. However the corrosion kinetics decreased with the number of wet–dry cycles because a porous oxide layer formed at the steel surface. Additionally, corrosion products filled the carbonated area and remained localized around the crack. L’Hostis states that carbonation-induced corrosion at the location of a crack would not be the most harmful parameter due to a possible reinforcement depassivation for the structure’s sustainability. To summarize, the limit state of depassivation for the whole structure, including its cracks, is a conservative approach. It is even more conservative than accounting only for uncracked concrete.

## 7. Conclusions

In this study, four extensions for the *fib* carbonation model to account for cracks in concrete are presented, applied, and discussed. To validate these model extensions, a comprehensive dataset collected from the literature, as well as newly acquired experimental data, were utilized. The following conclusions can be made:

- Both CIF approaches had a similar good prediction accuracy. The carbonation depth for concrete with natural cracks was predicted well. However, the models tend to underestimate the carbonation depth with increasing exposure time.
- The diffusion-based model significantly underestimated carbonation depths for experiments with low ${\mathrm{CO}}_{2}$ concentrations.
- The crack depth adaption overestimated the carbonation depth in (nearly) all cases. For natural cracks, the carbonation depth was even overestimated by up to a factor 3.5. However, the prediction accuracy increased with increasing exposure time.

The findings of this study highlight the limitations of the presented engineering models for cracked carbonated concrete. In the future, high-fidelity multi-physics models should be used instead of engineering models to have more accurate predictions of the carbonation depth in cracked concrete and to realistically account for the influence of crack geometric factors on carbonation.

A theoretical case study for reinforced concrete with a 35 mm concrete cover was carried out. The cracks were assumed to reach the reinforcement on average. The predicted probability of depassivation after 50 years were:

- 0% for the uncracked concrete structure.
- Significantly below 1% for the whole structure, which includes the 0.65% cracked surface. However, the probability of depassivation could be underestimated because the carbonation from the crack surface and from the crack along the steel–concrete interface is not accounted for. Additionally, while such reliability assessments predict low depassivation probabilities, cracks can speed up local corrosion with potential consequences for the entire structure.
- Between 1% and 70% in the cracked area depending on the modeling approach. Structural investigations indicate that the depassivation probability of about 1% predicted by the CIF approaches is unrealistically low. In contrast, the 70% probability of depassivation of the other approaches highlights the risk of potential damage at the location of a crack. However, preventing this higher probability of depassivation could make reinforced concrete structures cost-inefficient. An alternative approach is to change the limit state for the reliability assessment from the very conservative depassivation limit (initiation period) to the corrosion propagation state.

## Figures and Tables

**Table 1:** Overview of studies with accelerated carbonation tests with pre-cracked concrete from literature.

Parameter	Unit	Van Mullem et al. [13]		De Schutter [14]		Zhang et al. [10]	Guo et al. [37]
mix		mortar	mortar	mortar	mortar	concrete	paste
binder		50 w.% CEM I 52.2 N, 50 w.% fly ash	50 w.% CEM I 52.2 N, 50 w.% fly ash	CEM I 42.5 R	85 w.% CEM I, 15 w.% fly ash	OPC	OPC 42.5
crack type		notch	natural	notch	notch	notch	notch
uncracked concrete tested		✔	✔	✔	✔	✔	✔
*w*	[mm]	0.1, 0.2, 0.3	0.1, 0.2, 0.3	0.5	0.5	0.1, 0.2, 0.3, 0.5, 0.62	0.1, 0.2,0.3
*d*	[mm]	30	50	10	10	20	10, 15, 20, 25, 30
${\mathrm{CO}}_{2}$	[Vol.%]	1	1	10	10	20	20
$t_{exposure}$	[days]	14–63	14–31	56–196	56–196	7–56	7–21
$k_{e}$	[−]	1.11	1.11	1	1	0.86	0.86
$k_{c}$	[−]	0.32	0.32	0.45	0.45	0.46	0.46

**Table 2:** XRF results of used ordinary Portland cement (CEM I 42.5 R) and fly ash in [w.%].

Binder	CaO	SiO_2_	Al_2_O_3_	Fe_2_O_3_	MgO	K_2_O	Na_2_O	TiO_2_	P_2_O_5_	MnO	LIO	Na_2_O_eq_	Others
CEM I 42.5 R	57.90	18.85	5.43	3.33	2.30	1.64	0.06	0.24	0.12	0.08	3.44	1.14	
Fly ash	3.14	48.09	29.66	9.16	0.07	1.27	0.43	3.4	0.31	1.27	1.75		1.45

**Table 3:** Input parameters used for the comparison of the models for the dataset collected.

Parameter	Unit	Van Mullem et al. [13]	De Schutter [14]		Zhang et al. [10]	Guo et al. [37]		
$R_{ACC}^{-1}$	[$\frac{{\mathrm{mm}}^{2}/\mathrm{year}}{{\mathrm{kgCO}}_{2}/m^{3}}$]	127,166	234	451	1676	3015	6308	16,994
RH	[%]	60	65	65	70	70	70	70
$t_{c}$	[days]	51	28	28	28	28	28	28
ToW	[−]	0	0	0	0	0	0	0
$p_{SR}$	[−]	0	0	0	0	0	0	0
$C_{s}$	[$\frac{{\mathrm{kgCO}}_{2}}{m^{3}}$]	0.018	0.182	0.182	0.367	0.367	0.367	0.367
*C*	[$\frac{\mathrm{kg}}{m^{3}}$]	486 ^1^	499 ^1^	499 ^1^	400	1590 ^1^	1742 ^1^	1371 ^1^
CaO	[−]	30.52 ^2^	57.90 ^2^	49.69 ^2^	66.60	62.24	62.24	62.24
w/b	[−]	0.55	0.5	0.5	0.5	0.3	0.35	0.4
α	[−]	0.93 ^3^	0.91 ^3^	0.91 ^3^	0.80 ^3^	0.71 ^3^	0.78 ^3^	0.83 ^3^

^1^ Calculated with $\rho_{binder}=3040$ [$\frac{\mathrm{kg}}{m^{3}}$], $\rho_{aggregates}=2550$ [$\frac{\mathrm{kg}}{m^{3}}$], $\rho_{water}=1000$ [$\frac{\mathrm{kg}}{m^{3}}$]. ^2^ Estimated with OPC 57.9 w.% CaO and fly ash to 3.14 w.% CaO. ^3^ Calculated with Equation (17).

**Table 4:** Input parameters used for the model’s validation from the experimental study.

Parameter	Unit	OPC	OPC + FA
$R_{ACC}^{-1}$	[$\frac{{\mathrm{mm}}^{2}/\mathrm{year}}{{\mathrm{kgCO}}_{2}/m^{3}}$]	1024	3407
RH	[%]	65	65
$t_{c}$	[days]	7	7
ToW	[−]	0	0
$p_{SR}$	[−]	0	0
$C_{s}$	[$\frac{{\mathrm{kgCO}}_{2}}{m^{3}}$]	0.036	0.036
*C*	[$\frac{\mathrm{kg}}{m^{3}}$]	499 ^1^	499 ^1^
CaO	[−]	57.90	46.95
w/b	[−]	0.5	0.5
α	[−]	0.91 ^2^	0.91 ^2^

^1^ Calculated with $\rho_{binder}=3040$ [$\frac{\mathrm{kg}}{m^{3}}$], $\rho_{aggregates}=2550$ [$\frac{\mathrm{kg}}{m^{3}}$], $\rho_{water}=1000$ [$\frac{\mathrm{kg}}{m^{3}}$]. ^2^ Calculated with Equation (17).

**Table 5:** Input parameters of the theoretical probabilistic case study.

Parameter	Unit	Distribution	Mean	Std	Boundaries
$R_{ACC,0}^{-1}$	[$\frac{{\mathrm{mm}}^{2}/\mathrm{year}}{{\mathrm{kgCO}}_{2}/m^{3}}$]	Normal distribution	2144 ^1^	273.6 ^1^	
RH	[%]	Beta distribution	71.96	20.92	[0, 100]
$t_{c}$	[days]		7		
ToW	[−]		77/365		
$p_{sr}$	[−]	Beta distribution	0.3	0.1	[0, 1]
$C_{s}$	[$\frac{{\mathrm{kgCO}}_{2}}{m^{3}}$]	Normal distribution	0.00082 ^1^	0.0001 ^1^	
*C*	[$\frac{\mathrm{kg}}{m^{3}}$]		280		
CaO	[−]		0.579		
α	[−]		0.91		
$c_{nom}$	[mm]	Beta distribution	35	6 ^1^	[0, 175] ^1^
*w*	[mm]	Normal distribution	0.25	0.015	
*d*	[mm]	Beta distribution	35	5	[0, 175]

^1^ according to *fib* MC SLD [1].

## Data Availability

The dataset containing input parameters and model output, as well as the validation dataset, are available for download: https://github.com/KIT-IMB-Modeling-And-Digitalization/data_carbonation_cracked_concrete (accessed on 7 September 2023).

## References

[1] International Federation for Structural Concrete (2006). Model Code for Service Life Design.

[2] International Federation for Structural Concrete (2012). Model Code 2010: Final draft. Bulletin/International Federation for Structural Concrete Draft Model Code.

[3] ACI Committee 224 (1997). 224.2R-92 Cracking of Concrete Members in Direct Tension.

[4] Setareh M., Darvas R. (2017). Concrete Structures.

[5] (2010). Eurocode 2: Bemessung und Konstruktion von Stahlbeton-und Spannbetontragwerken—Teil 1-1: Allgemeine Bemessungsregeln und Regeln für den Hochbau.

[6] Mehta P.K., Monteiro P.J.M. (2006). Concrete: Microstructure, Properties, and Materials; [CD-ROM with 1000+ Powerpoint Slides, Videos, Bonus Material, and More.

[7] Wu L., Farzadnia N., Shi C., Zhang Z., Wang H. (2017). Autogenous shrinkage of high performance concrete: A review. Constr. Build. Mater..

[8] Safiuddin M., Kaish A., Woon C.O., Raman S. (2018). Early-Age Cracking in Concrete: Causes, Consequences, Remedial Measures, and Recommendations. Appl. Sci..

[9] Al-Ahmad S., Toumi A., Verdier J., François R. (2009). Effect of crack opening on carbon dioxide penetration in cracked mortar samples. Mater. Struct..

[10] Zhang S.P., Zong L., Dong L.F., Zhang W. (2011). Influence of Cracking on Carbonation of Cement-Based Materials. Adv. Mater. Res..

[11] Han J., Liu W., Wang S., Du D., Xu F., Li W., de Schutter G. (2016). Effects of crack and ITZ and aggregate on carbonation penetration based on 3D micro X-ray CT microstructure evolution. Constr. Build. Mater..

[12] Bogas J.A., Carriço A., Pontes J. (2019). Influence of cracking on the capillary absorption and carbonation of structural lightweight aggregate concrete. Cem. Concr. Compos..

[13] van Mullem T., de Meyst L., Handoyo J.P., Caspeele R., de Belie N., van den Heede P. (2020). Influence of Crack Geometry and Crack Width on Carbonation of High-Volume Fly Ash (HVFA) Mortar. Proceedings of the 3rd RILEM Spring Convention and Conference (RSCC 2020).

[14] De Schutter G. (1999). Quantification of the influence of cracks in concrete structures on carbonation and chloride penetration. Mag. Concr. Res..

[15] AL-Ameeri A., Rafiq M.I., Tsioulou O. Influence of cracks on the carbonation resistance of concrete structures. Proceedings of the Sixth International Conference on the Durability of Concrete Structures-ICDCS 2018.

[16] Bogas J.A., Ahmed H.H., Diniz T. (2021). Influence of Cracking on the Durability of Reinforced Concrete with Carbon Nanotubes. Appl. Sci..

[17] Varzina A., Phung Q.T., Perko J., Jacques D., Maes N., Cizer Ö. (2022). Synergistic Effects between Carbonation and Cracks in the Hardened Cement Paste. Sustainability.

[18] Schiessl P. (1976). Zur Frage der zulässigen Rißbreite und der erforderlichen Betondeckung im Stahlbetonbau unter besonderer Berücksichtigung der Karbonatisierung des Betons. Schriftenreihe des Deutschen Ausschusses für Stahlbeton.

[19] Sullivan-Green L., Hime W., Dowding C. (2007). Accelerated protocol for measurement of carbonation through a crack surface. Cem. Concr. Res..

[20] Kwon S.J., Na U.J. (2011). Prediction of Durability for RC Columns with Crack and Joint under Carbonation Based on Probabilistic Approach. Int. J. Concr. Struct. Mater..

[21] Häkkinen T. (1993). Influence of High Slag Content on the Basic Mechanical Properties and Carbonation of Concrete. Ph.D. Thesis.

[22] (2007). China Engineering Construction Standardization Association Professional Committee of identification and reinforcement of buildings, Standard for durability assessment of concrete structures.

[23] Guiglia M., Taliano M. (2013). Comparison of carbonation depths measured on in-field exposed existing r.c. structures with predictions made using fib-Model Code 2010. Cem. Concr. Compos..

[24] Yang K.H., Seo E.A., Tae S.H. (2014). Carbonation and CO_2_ uptake of concrete. Environ. Impact Assess. Rev..

[25] Silva A., Neves R., de Brito J. (2014). Statistical modelling of carbonation in reinforced concrete. Cem. Concr. Compos..

[26] Hills T.P., Gordon F., Florin N.H., Fennell P.S. (2015). Statistical analysis of the carbonation rate of concrete. Cem. Concr. Res..

[27] von Greve-Dierfeld S., Gehlen C. (2016). Performance based durability design, carbonation part 1—Benchmarking of European present design rules. Struct. Concr..

[28] Ekolu S.O. (2018). Model for practical prediction of natural carbonation in reinforced concrete: Part 1—formulation. Cem. Concr. Compos..

[29] ossan E., Andrade J.J.O., Dal Molin D.C.C., Ribeiro J.L.D., Delgado J. (2021). Model to Estimate Concrete Carbonation Depth and Service Life Prediction. Hygrothermal Behaviour and Building Pathologies.

[30] Bentz D.P., Garboczi E.J., Lu Y., Martys N., Sakulich A.R., Weiss W.J. (2013). Modeling of the influence of transverse cracking on chloride penetration into concrete. Cem. Concr. Compos..

[31] Peng J., Hu S., Zhang J., Cai C.S., Li L.y. (2019). Influence of cracks on chloride diffusivity in concrete: A five-phase mesoscale model approach. Constr. Build. Mater..

[32] Liang M.T., Qu W.J., Liao Y.S. (2000). A study on carbonation in concrete structures at existing cracks. J. Chin. Inst. Eng..

[33] Schiessl P., Comité euro-international du béton (1997). New approach to durability design: An example for carbonation induced corrosion. Bulletin d’information/Comité Euro-International du Béton.

[34] Schultheiß A., Patel R.A., Vogel M., Dehn F. (2022). Comparative study of probabilistic modelling approaches for chloride ingress in concrete structures with macro-cracks. Struct. Concr..

[35] Song H.W., Kwon S.J., Byun K.J., Park C.K. (2006). Predicting carbonation in early-aged cracked concrete. Cem. Concr. Res..

[36] Carević V., Ignjatović I. (2019). Influence of loading cracks on the carbonation resistance of RC elements. Cem. Concr. Res..

[37] Guo Q., Jiang L., Wang J., Liu J. (2022). Analysis of Carbonation Behavior of Cracked Concrete. Materials.

[38] Bejaoui S., Bary B. (2007). Modeling of the link between microstructure and effective diffusivity of cement pastes using a simplified composite model. Cem. Concr. Res..

[39] (2009). Prüfverfahren für Zement_- Teil_1: Bestimmung der Festigkeit.

[40] Deutscher Wetter Dienst Climate Data Center (CDC). https://opendata.dwd.de/climate_environment/CDC/observations_germany/climate/.

[41] Francois R., Maso J.C. (1988). Effect of damage in reinforced concrete on carbonation or chloride penetration. Cem. Concr. Res..

[42] Dang V.H., François R., L’Hostis V. (2013). Effects of pre-cracks on both initiation and propagation of re-bar corrosion in pure carbon dioxide. EPJ Web Conf..

[43] Ghantous R.M., Poyet S., L’Hostis V., Tran N.C., François R. (2017). Effect of crack openings on carbonation-induced corrosion. Cem. Concr. Res..

[44] L’Hostis V. (2020). Long-term corrosion of rebars submitted to concrete carbonation. Mater. Corros..

